# Potential anticancer properties and mechanisms of thymoquinone in colorectal cancer

**DOI:** 10.1186/s12935-023-03174-4

**Published:** 2023-12-12

**Authors:** Farhad Sheikhnia, Vahid Rashidi, Hossein Maghsoudi, Maryam Majidinia

**Affiliations:** 1 Student Research Committee, Urmia University of Medical Sciences, Urmia, Iran; 2 Department of Clinical Biochemistry, School of Medicine, Urmia University of Medical Sciences, Urmia, Iran; 3 Solid Tumor Research Center, Cellular and Molecular Medicine Institute, Urmia University of Medical Sciences, Urmia, Iran

**Keywords:** CRC, Colorectal cancer, Anti-cancer, Apoptosis, Signaling pathway, Thymoquinone

## Abstract

Colorectal neoplasms are one of the deadliest diseases among all cancers worldwide. Thymoquinone (TQ) is a natural compound of *Nigella sativa* that has been used in traditional medicine against a variety of acute/chronic diseases such as asthma, bronchitis, rheumatism, headache, back pain, anorexia, amenorrhea, paralysis, inflammation, mental disability, eczema, obesity, infections, depression, dysentery, hypertension, gastrointestinal, cardiovascular, hepatic, and renal disorders. This review aims to present a detailed report on the studies conducted on the anti-cancer properties of TQ against colorectal cancer, both in vitro and in vivo. TQ stands as a promising natural therapeutic agent that can enhance the efficacy of existing cancer treatments while minimizing the associated adverse effects. The combination of TQ with other anti-neoplastic agents promoted the efficacy of existing cancer treatments. Further research is needed to acquire a more comprehensive understanding of its exact molecular targets and pathways and maximize its clinical usefulness. These investigations may potentially aid in the development of novel techniques to combat drug resistance and surmount the obstacles presented by chemotherapy and radiotherapy.

**Graphical Abstract:**

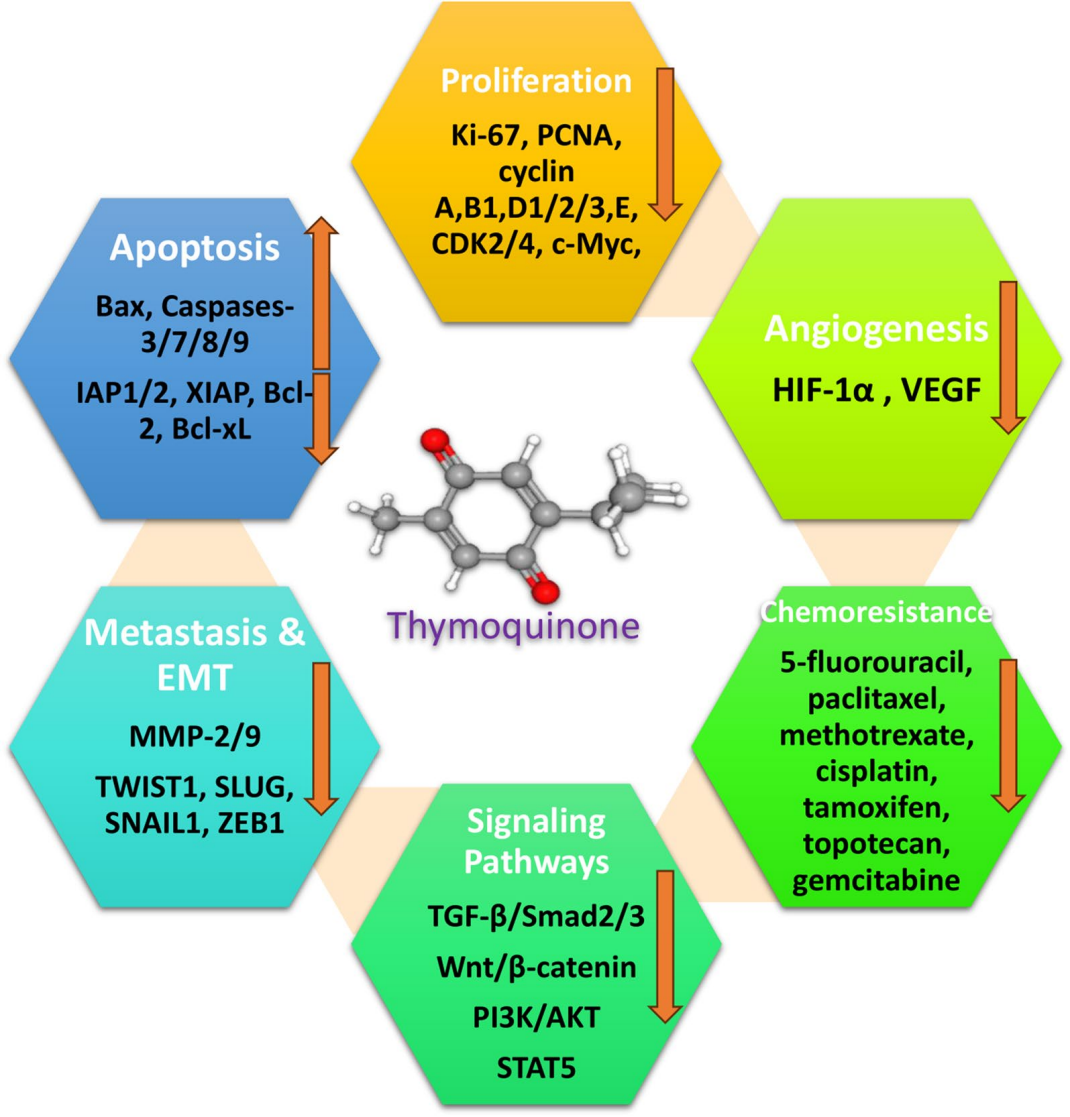

## Introduction

According to the GLOBOCAN report, colorectal cancer is the third most common cancer and the second leading cause of death among all cancers globally, affecting both genders [1]. In the year 2020, the estimated quantity of fresh instances of colorectal cancer was 1.9 million, leading to 930,000 fatalities. These statistics are estimated to increase to 3.2 million fresh instances and 1.6 million fatalities by the year 2040 [2]. The endangerment variables correlated with colorectal cancer involve an inactive way of life (obesity), ingestion of a diet high in crimson flesh, alcohol, and low fiber foods, smoking, and chronic inflammation. Furthermore, genetic predisposition has been determined to be a probable cause for the emergence of colorectal cancer [3, 4]. Aging is another risk factor for colorectal cancer (CRC) since 90 percent of new incidences occurs in 50 years old or later [4]. Moreover, men have higher risk (1.3-fold) for CRC than women [5]. The significance of screening for CRC cannot be emphasized enough since it has the potential to identify precancerous polyps as well as early-stage cancers at the most opportune time for treatment. Colonoscopy, fecal occult blood testing, and stool DNA testing are some of the existing screening methods available [6]. According to the American Cancer Society, individuals who are at average risk of developing CRC should start screening at age 45 with promptness.

Preventing CRC onset can be achieved by inducing lifestyle changes such as maintaining a nutrient-rich diet, keeping excess weight off, participating in activities that encourage physical movement, as well as avoiding the use of tobacco products and excess alcohol intake. Moreover, using nonsteroidal anti-inflammatory drugs (NSAIDs) for chemoprevention purposes has been known to effectively lower the risk of developing CRC [7].

5-fluorouracil, capecitabine, oxaliplatin, irinotecan, and monoclonal antibodies such as bevacizumab, panitumumab, and cetuximab are well-known chemotherapy agents administered as monotherapy or combination therapy [8–10]. Despite the positive effects of the aforementioned agents in CRC control and treatment, most patients complain of neutropenia, vomiting, diarrhea, neurotoxicity, and mucositis which are attributed to especially higher doses of these drugs [11].

*Nigella sativa*, also known as black seed or black cumin, is an annual herb in the Ranunculacea family with numerous medicinal properties [12]. It contains a variety of active ingredients, including TQ, alkaloids, saponins, flavonoids, proteins, and fatty acids and these components have been shown to have positive effects in treating various diseases [13]. Preclinical studies have demonstrated its potential for use as an anti-cancer, antimicrobial, analgesic, antipyretic, contraceptive and anti-fertility, anti-oxytocic, anti-tussive, anti-inflammatory and antioxidant agent [13]. This review aims to present a detailed report on the studies conducted on the anti-cancer properties of thymoquinone (TQ) against CRC, both in vitro and in vivo.

## Colorectal cancer pathogenesis

The development of colorectal cancer is a complex process that is influenced by a combination of genetic and environmental factors and involves the accumulation of genetic alterations in normal colonic mucosa. These alterations can include mutations in genes such as adenomatous polyposis coli (APC), KRAS, TP53, and SMAD4 [14]. APC mutations, which are found in up to 80% of sporadic colorectal cancer cases, are considered an early event in the pathogenesis of the disease [14]. APC is a tumor suppressor gene that regulates cell proliferation and differentiation. When mutated, it can lead to the activation of the Wnt signaling pathway, promoting cell proliferation and inhibiting apoptosis [7]. KRAS mutations, found in approximately 40% of colorectal cancer cases, are associated with a poor prognosis. KRAS is a proto-oncogene that regulates cell growth and differentiation. When mutated, it can lead to the activation of downstream signaling pathways that promote cell proliferation and survival [7, 14]. TP53 mutations, found in approximately 50% of colorectal cancer cases, are also associated with a poor prognosis. TP53 is a tumor suppressor gene that regulates cell cycle arrest, DNA repair, and apoptosis. When mutated, it can lose its tumor suppressor function, promoting cell proliferation and inhibiting apoptosis. SMAD4 mutations, found in approximately 10% of colorectal cancer cases, are associated with a poor prognosis as well. SMAD4 is a tumor suppressor gene that regulates TGF-β signaling. When mutated, it can lead to the activation of downstream signaling pathways that promote cell proliferation and survival [7, 14].

Epigenetic alterations, which are changes in gene expression that do not involve changes to the DNA sequence, play a critical role in the development of colorectal cancer. The two most common epigenetic alterations in this disease are DNA methylation and histone modification. DNA methylation involves the addition of methyl groups to cytosine residues in CpG dinucleotides. When CpG islands located in the promoter regions of tumor suppressor genes become hypermethylated, it can lead to the silencing of these genes and the loss of their tumor suppressor function. This can promote cell proliferation and inhibit apoptosis [15]. Histone modification refers to changes in the structure of chromatin that can affect gene expression. Histones can be modified through processes such as acetylation, methylation, phosphorylation, or ubiquitination. These modifications can either activate or repress gene expression depending on their location within the chromatin structure [15].

The escalation from colorectal adenoma to carcinoma is caused by three cardinal pathways: microsatellite instability (MSI), chromosomal instability (CIN), and CpG island methylator phenotype (CIMP) [16]. When there is a malfunction in the DNA mismatch repair genes, MSI occurs, leading to an amassment of transformations in microsatellite areas throughout the genome. MSI is perceived in approximately 15% of sporadic CRC cases and is correlated with a superior prognosis [16]. On the other hand, CIN occurs due to aneuploidy or chromosomal rearrangements, leading to a buildup of genetic changes throughout the genome. CIN is identified in approximately 85% of sporadic CRC cases and is related to an adverse prognosis [16]. Finally, CIMP is identified upon hypermethylation of CpG islands situated in promoter regions of tumor suppressor genes, leading to their suppression. CIMP is identified in approximately 20% of sporadic CRC cases and is correlated with an unfavorable prognosis [16].

## Anticancer effects of TQ

*Nigella sativa L.* (also known as black cumin, black seed, and black caraway) is a spice that also has been used in traditional medicine against a variety of acute/chronic diseases such as asthma, bronchitis, rheumatism, headache, back pain, anorexia, amenorrhea, paralysis, inflammation, mental disability, eczema, obesity, infections, depression, dysentery, hypertension, gastrointestinal, cardiovascular, hepatic, and renal disorders based on antimicrobial/viral, antioxidative, anti-diabetic, anti-inflammatory, and immunomodulatory properties of its components specifically TQ [17–24].

TQ is the main bioactive component of the volatile oil of *N. sativa* [18, 25–27]. Other components of *N. sativa* L. are thymohydroquinone, thymol, carvacrol, nigellidine, nigellicine, and α-hederin [28, 29]. Thanks to chemo preventive and anti-tumor properties, TQ and *N. sativa* has been used against various neoplasms including lung, hepatobiliary, liver, breast, pancreas, hematopoietic/leukemia, kidney, bladder, cervix, skin, ovary, prostate, osteosarcoma, fibrosarcoma, and colorectal cancers in-vivo and in-vitro with less cytotoxicity against normal cells [18, 20, 22, 25, 30]. Different studies showed that TQ could target various mechanisms involved in cancer progression including proliferation [21, 22, 24, 30–38], migration [21, 30, 32, 34–36, 39–41] invasion/metastasis [22, 30, 32, 35, 41, 42], angiogenesis [22, 31, 41], colony formation [35, 36], tubulogenesis [43, 44], epithelial-mesenchymal transition (EMT) [32, 34, 42, 45], autophagy [45–47], and cancer stemness [43].

Furthermore, TQ enhanced tumor cell cytotoxicity [21, 43, 48], cell cycle arrest at G2/M [49–55], G1/S [19, 31, 56, 57], S [58], and G0/G1 [30, 38, 42, 48, 50, 59], apoptosis [19, 22, 30, 31, 35, 37, 38, 48, 56, 60–65], and necroptosis [66].

A vast variety of proteins and signaling pathways that play key roles in cancer pathogenesis are reported to be modulated by TQ including inhibition of E2F-1 [67], eEF-2 K [21], microphthalmia‑associated transcription factor (MITF) [33], Rac1 [40], Notch1 [68], Src/FAK [21, 31, 69–71], PI3K/Akt/mTOR [21, 31, 34, 36, 48, 72, 73], TGF‑β/Smad2/3 [32], Wnt/β-catenin [33, 42], tubulin α/β [74], NF-κB and p65 [21, 22, 31], TNF-α [75], anti-apoptotic proteins (IAP1/2, XIAP, Bcl-2, Bcl-xL Mcl-1, c-FLIP and survivin) [22, 30, 31, 48, 50, 62, 63, 76], PD-L1 [45, 64], HIF-1α [77], MUC4 [69, 78], ENA-78 and Gro [79], androgen receptor [67], Plk1 [80], IRAK1 [81], proliferative proteins (cyclin A, cyclin B1, cyclin D1/2/3, cyclin E, CDK2/4, c-Myc, Ki-67, PCNA) [22, 31, 42, 48, 50, 57, 65, 67, 73, 82, 83], CXCR4 [84, 85], Integrin-β1 [47], Beclin-1 and LC3 [47], COX-2 [22, 84], HSP70 [58], u-PA [86], MMP-2/9 [22, 39, 70], MMP-3/7 [42, 87], Trx1 [88], VEGF [22, 31, 39], ERK1/2 [41, 70, 89], JNK [71, 90], NLRP3 [91], BCR-ABL [37], JAK2/STAT3 [31, 37, 65, 72, 73], FLT3-ITD [38], IL-8 [54], STAT5a/b [37, 72, 73], EP2 [92], vimentin, TWIST1, SLUG, SNAIL1, ZEB1, and N-cadherin [32, 34, 45], and promoting expression of SH-PTP2 [31], SHP-1 [38, 93], SOCS-1/3 [38, 93], p16 [50], p21 (CIP1/WAF1) [51, 67], p27 (Kip1) [48, 67], activated p38/MAPK [61, 69], p53 [40, 48, 50], p62 [46], p73α/β [59, 74], PPAR-γ [30], PTEN [52, 72], PKM2 [94], Bax [48, 62, 63, 95], LKB1/AMPK [96], Par-4 [97], Bad [48], LC3-II [46], E-cadherin [32, 34, 45, 98], Tristetraprolin (TTP) [78], gelsolin [99], TIMP3 [39], HSPA6 [100], Nrf2 [64], IL17RD [101], cleavage of caspases-3/7/8/9, and PARP [30, 31, 36, 48, 61, 63, 95], TRAIL [102], DR5 (TRAIL-R2) [54, 102, 103], cytochrome C release [48, 95] and ROS production/oxidative stress [54, 59, 65, 104].

Hypermethylation of tumor suppressor genes which leads to the downregulation of these TSGs contributes to several malignancies [38] Epigenetic modulation is another mechanism of action of TQ to combat cancers, in this regard, expression of SHP-1 and SOCS-3, two TSGs inhibiting JAK/STAT pathway, increased through TQ-induced hypomethylation of CpG island of these genes’ promoters [38, 93]. This modulation was considered to be related with TQ-induced upregulation of TET2 and WT1 and downregulation of DNA methyltransferases 1/3A/3B, UHRF1, HDAC1/2 [38, 59, 88, 105].

Several studies indicated that TQ exerted its anti-cancer characteristics via non-coding RNAs such as upregulation of miR-603 [21] and miR-1-3p [39], miR-34a [40, 106, 107], miR-125a-5p [108], miR-16 [106, 109], miR-877-5p [45], and miR-375 [109].

In-vitro studies also mentioned the combination of TQ with anti-neoplastic agents or flavonoids such as thalidomide [31], temozolomide [110], bortezomib [31], doxorubicin [30], 5-fluorouracil [30, 111], paclitaxel [30], methotrexate [62, 63], cisplatin [79, 112], tamoxifen [113], topotecan [114], gemcitabine [68, 94], curcumin [36], quercetin [115], and emodin [116] augmented cytotoxic effects of these chemicals against cancer cells.

Interestingly, since TQ like other flavonoids and polyphenolic compounds are sensitive to light and pH, incorporation of TQ into various nanoparticles increased its solubility, stability, and improved its efficacy against tumor cells [117–119]. Various nanoparticles have been used in this manner including chitosan encapsulating poly D,L-lactic-co-glycolic acid (PLGA) [118], lipid [120], liposome [60, 121, 122], PEG [40], and PLGA [20], zinc oxide [123], mesoporous silica [124], and lipid polymer hybrid nanoparticles [125].

## TQ and colorectal cancer

**Fig. 1:**
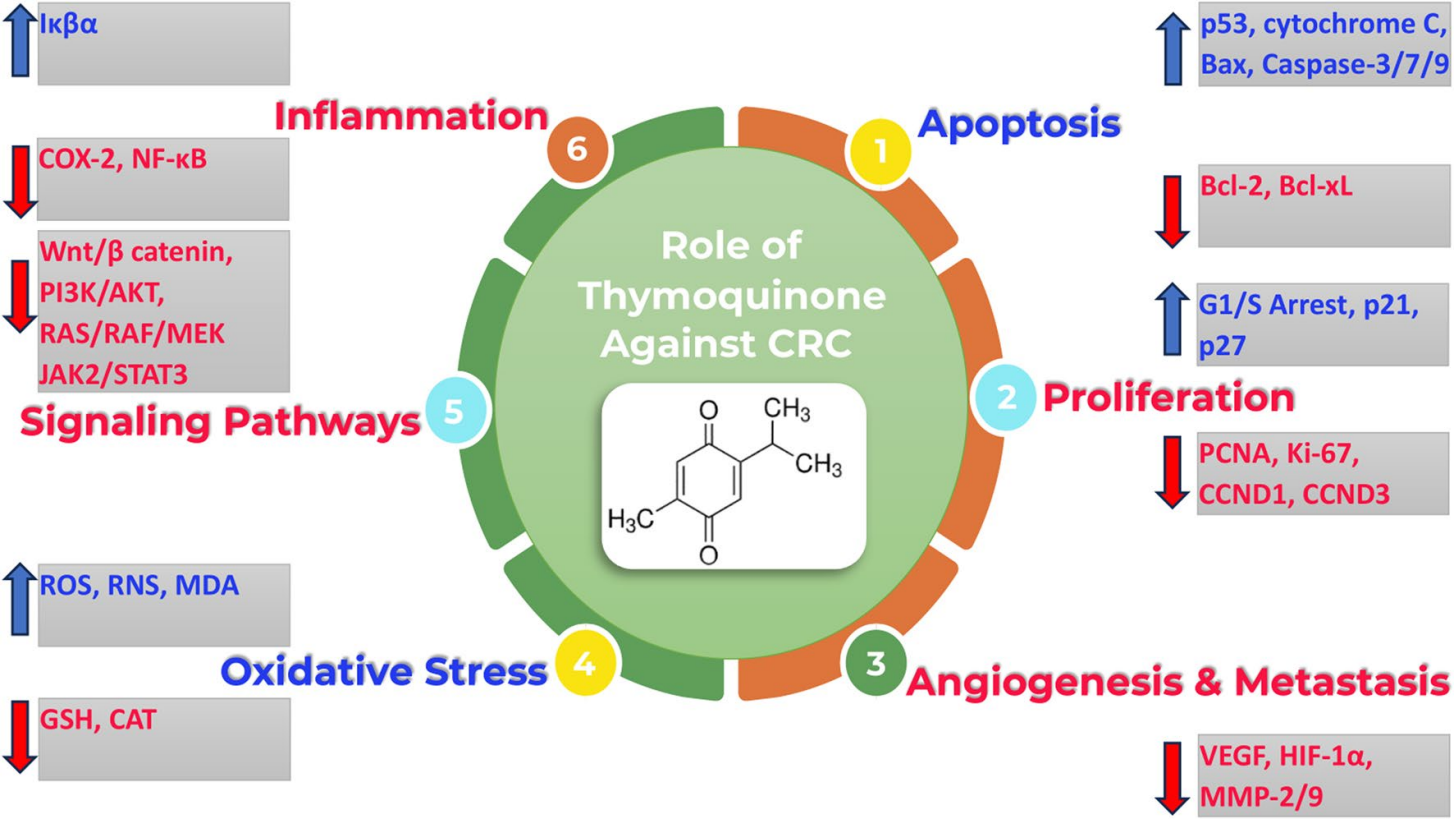
TQ, acting via multiple targets, may serve as a potential natural therapeutic agent against CRC. This is achieved through the augmentation of apoptosis and oxidative stress, coupled with the attenuation of cell cycle progression/proliferation, inflammation, CRC-associated signaling pathways, angiogenesis, and metastasis

A multitude of preclinical studies have been conducted in the realm of colorectal cancer research (Fig. 1). As previously discussed, TQ demonstrates a notable cytotoxic effect against cancer cells, while sparing normal cells [126]. In the study conducted by Eftekhar et al., it was observed that TQ significantly enhances the Area Under the Curve (AUC), Maximum Concentration (Cmax), and Time to Reach Maximum Concentration (Tmax) of 5-FU, thereby augmenting its pharmacokinetic profile. Interestingly, even trace amounts of TQ were observed to potentiate the growth-inhibitory effects of 5-FU on colorectal cancer cells. Furthermore, TQ was found to reduce the viability of HT-29 cells in a dose-dependent manner, with an IC50 value of 0.284 mM. Notably, the combined administration of TQ and 5-FU resulted in an enhanced cytotoxic effect compared to the individual suppressive impact of 5-FU. This combination demonstrated a significant suppressive effect at 5-FU concentrations of 0.027 and 0.055 mM, suggesting that TQ could potentially amplify the growth-suppressive effects of 5-FU on cancer cells [127]. The concurrent administration of 30 μM imatinib, a tyrosine kinase inhibitor, and 10 μM TQ resulted in the suppression of ABCB1, ABCG2, and hOCT1, thereby enhancing the uptake of imatinib in HCT-116 cells [128]. The combination of 20 and 40 µM TQ with 0.2 µM cisplatin amplified the cytotoxicity of cisplatin in HCT-116 and COLO205 cells. This data suggests that TQ potentiates cisplatin-induced cell death in a dose-dependent manner, indicating a potential role for TQ in augmenting the chemosensitivity of colon cancer cells [129]. Research conducted by Osorio-Pérez demonstrated a significant reduction in the expression levels of miR-21-5p in HCT-15 cells following TQ treatment [130]. Interestingly, the combination of TQ with ionizing radiation (IR) enhanced cytotoxicity against HT-29 and HCT-116 cells. The combination of a low dose of TQ (3 µM) with IR (2 Gy) resulted in complete inhibition of sphere formation by the fifth generation. This outcome was linked to the suppression of stemness and DNA repair mechanisms [131]. An in vivo study was conducted to evaluate the protective role of TQ against DMH-induced CRC in adult male Wistar rats. Both pre and post treatment with TQ significantly inhibited CRC initiation and progression. Notably, pre-treatment with TQ was more effective than post-treatment. The protective effects of TQ include reduced ROS production and lipid peroxidation (MDA) [132]. Moreover, TQ alone or in combination with vitamin D showed favorable outcomes in Azoxymethane-induced CRC rats [133] (see Table 1).

**Table 1:** Represents the experimental TQ studies on CRC in vitro and in vivo

Study	Cells; IC_50_	Animal model	Effects	Refs.
In vitro	HCT-116; 12 h: 60 µM		G1/S cell cycle arrest, enhanced apoptosis, increased p53 and p21, decreased proliferation, reduced Bcl-2	[146]
	HCT-116; 24 h: 35 µM			
	HCT-116; 48 h: 22 µM			
In vitro	HT-29; 24 h: 51.6 ± 3.0 µM HT-29; 48 h: 53.3 ± 1.7 µM HT-29; 72 h: 51 ± 0.7 µM		Enhanced necrosis, decreased proliferation	[148]
In vitro and In vivo	C26; 24 h: 40 μM	Female Balb/c mice	Enhanced apoptosis and decreased ACF (not proliferation) in vivo, decreased invasion in vitro	[150]
In vitro	HCT-116; 24/48 h: 30/14 μM LoVo; 24/48 h: 38/28 μM DLD-1; 24/48 h: 42/23 μM Caco-2; 24/48: 15/12.5 μM HT-29; 48 h:110 μM FHs74Int (Normal Cell Line)		Enhanced apoptosis (caspase-3) in DLD-1 (not HT-29), increased ROS in DLD-1 and Caco-2, decreased proliferation of all CRC cell lines (not FHs74Int)	[126]
In vitro and In vivo	DLD1; 196 μM HCT116; 118 μM RKO; 86 μM HCEC-1CT; 79 μM HT29; 160 μM LoVo; 36 μM	Apc^Min^ mice	Enhanced apoptosis in polyps, decreased proliferation (Ki-67) of villi, reduced c-myc expression in polyps and in vitro, RAS/RAF/MEK1/2 inhibition, Wnt/ β-catenin inhibition, suppressed GSK-3β phosphorylation	[143]
In vitro	HCT-116		Enhanced apoptosis (increased p21, p27, cleavage of caspases-3/7/9 and PARP), decreased activated form, JAK2/STAT3 inhibition, Src inhibition	[145]
In vitro	HCT-116; 72 h: GI50: 12.7 ± 0.9 μM HT-29; 72 h: GI50: 27.3 ± 3.0 μM		Enhanced apoptosis (mainly through late apoptotic process), increased NQO1 protein level, decreased (glutathione) GSH activity	[147]
In vitro	HCT-116; 24 h: 64.15 ± 2.80 µM		Enhanced apoptosis (increased caspase-3 activity/mRNA levels and Bax while decreased Bcl-2) in combination with Doxorubicin	[158]
In vitro and In vivo	HCT-116; 59.64 μM	Immunodeficient female NCr nude homozygous mice	Free TQ and TQ-NP decreased tumor volume/weight alone or with Doxorubicin	[136]
In vitro	HT-29		Increased mRNA/protein levels of PPAR-γ	[159]
In vitro & In vivo	HCT-116; 48/72 h: 40 µM HCT-116/5FU; 48 h: 60 µM	NOD-SCID mice	Enhanced apoptosis (increased p53 and p21 while reduced NF-κB, PCNA and p-MEK protein levels), suppressed CD44, decreased migration/invasion of 5-FU sensitive and resistant cells Decreased tumor growth (increased p53, p21, γ-H2AX, Iκβα, and reduced PCNA, NF-κB (p65), and p-MEK), suppressed CD44 in 5-FU sensitive xenograft mice	[160]
In vitro	Dox-treated HCT-116; 24 h: 66.75 ± 2.00 µM		Enhanced apoptosis	[161]
In vivo		Azoxymethane-induced CRC rats	Decreased ACF, Wnt, β-catenin, NF-κB and COX-2, while increased DKK-1 and CDKN1-A mRNA levels, decreased TGF-β1, COX-2, HSP-90 and VEGF protein levels	[133]
In vitro and In vivo	LoVo	Nude mice	Inhibited migration, decreased p-PI3K, p-Akt, p-GSK3β, β-catenin, COX-2, and LEF-1/TCF-4 in vitro, Decreased COX-2, β-catenin, and p-Akt in vivo	[141]
In vitro	COLO205 HCT116		Increased chemosensitivity to cisplatin, Suppressed NF-κB p65 phosphorylation, VEGF, c-Myc, and Bcl-2 protein levels,	[129]
In vivo		AOM-induced CRC rate	Increased DKK-1, CDNK-1A, TGF-β1, and Smad4, suppressed Wnt, β-catenin, NF-κB, and COX-2 mRNA levels	[162]
In vitro	HT-29; 24 h: 59.2 µM HT-29; 48 h: 68.4 µM		Enhanced apoptosis, decreased proliferation	[163]
In vitro	CPT-11-R LoVo; 24 h: 6–8 µM		Increased unphosphorylated BAD, and reduced phosphorylated BAD increased autophagic cell death (upregulated Atg5, Atg7, Atg12, Beclin-1, LAMP2, LC3-II, and SQSTM1/p62, while downregulated Atg3), decreased IKKα/β and NF-κB, suppressed EMT and metastasis (decreased Snail, Twist, vimentin, MMP-2/9), inhibited ERK1/2 and PI3K, enhanced JNK and p38	[164, 165]
In vitro	HCT-116; 24 h: 21.71 µM HCT-116; 48 h: 20.53 µM SW480; 24 h: 10.26 µM SW480; 48 h: 10.50 µM		Decreased colony formation, proliferation, EMT, migration and invasion Reduced glucose fermentation, lactate production, and ATP production, Suppressed HexoKinase 2, PI3K/Akt, E-cadherin, increased N-cadherin	[139]
In vitro	HT29 SW480 SW620		Enhanced apoptosis, p21, p27, PTEN, BAX, Cytochrome-C, Caspase-3 Reduced proliferation, CCND1, CCND3, BCL-2, suppressed PI3K/AKT/mTOR	[140]
In vitro	HT29 SW480 SW620		Enhanced G2/M cell cycle arrest apoptosis, reduced CCND1, CCND3, PCNA, survivin, HIF1α, LDHA, and PDHK1, inhibited PI3K/AKT/mTOR, RAPTOR, and RICTOR, augmented p21, p27, BAX, Cytochrome-C, cleaved Caspase-3, PTEN, AMPKα, and PDH, increased ROS/RNS, MDA, and PCC, while reduced total GSH and catalase (CAT)	[61]
In vitro	HCT-15; 24 h: 82.59 μM		Reduced proliferation, Bcl-2, and miR-21-5p expression	[130]

TQ-loaded polymeric nanocapsules were synthesized by Ramzy et al., utilizing the nanoprecipitation technique, with Eudragit S100 serving as the polymeric shell. Anisamide was conjugated as a targeting ligand for sigma receptors, which are overexpressed by colon cancer cells. The anisamide-targeted nanocapsules exhibited superior cytotoxicity compared to non-conjugated nanocapsules and free TQ against HT-29 cells following 48 h of incubation. This increased cytotoxicity can be attributed to the high level of sigma receptor expression on HT-29 cells, leading to enhanced uptake of nanocapsules [134]. One study suggested that TQ has the potential to enhance replication fidelity and that the chemo preventive effects of TQ in Lynch syndrome are due to this property. TQ has been observed to decrease the incidence and multiplicity of intestinal tumors in Msh2 ^loxP/loxP^ Villin-Cre mice as well as MSI in Msh2-deficient epithelium [135]. Nano formulations of TQ with PLGA and PVA also enhanced the efficiency of TQ in HCT-116 xenograft models and also showed protective effects against doxorubicin-induced cardiotoxicity [136]. Encapsulated TQ in lipid nanocapsules (LNCs) enhanced its specificity and cellular absorption. In vivo studies revealed that intratumoral administration of TQ-LNCs led to a reduction in tumor size in mice with colorectal cancer, compared to the control group. Interestingly, TQ-LNCs proved to be more effective than free TQ in inducing tumor cell death [137].

### TQ targets signaling pathways

In a study conducted by El-Baba et al., an experiment was performed on colorectal HCT116wt cells using the PepChip Kinomics v2 peptide array. Following treatment with 40 μM TQ for a duration of 24 h, a significant increase in phosphorylation was observed in 104 proteins. Out of these proteins, 50 proteins and kinases exhibited an upregulation of ≥ twofold (out of 1152 kinase substrate peptides). Further analysis revealed that among the top 50 candidate proteins, 24 were classified into the cancer-related networks “cytoskeleton”, “PI3K/AKT”, and “Wnt signaling”. Upon the introduction of TQ, significant structural alterations were observed in P21-Activated Kinase 1 (PAK1), which disrupted its scaffold function in the pro-survival PAK1/MEK/ERK1/2 signaling pathway. This led to the modification of several signaling mechanisms: The binding affinity between ERK1/2 and PAK1 is enhanced, which inhibits the phosphorylation of pPAK1Thr212 by ERK1/2. This results in an increase in phosphorylation at the Thr423 site, which interferes with the catalytic domain of PAK1 and prevents PAK1 activation. Ultimately, this cascade of events leads to the induction of apoptosis [138].

The PI3K/Akt signaling pathway is frequently implicated in the progression of CRC [139]. TQ has been observed to inhibit PI3K/Akt activation in CRC cell lines, including HCT-116 and SW480 probably through enhancing PTEN tumor suppressor [61, 139, 140]. This inhibition could potentially alter metabolic reprogramming in CRC cells, as evidenced by the suppression of factors and enzymes related to glycolysis and the Warburg effect, including HIF1α, hexokinase2, PDHK1, and LDHA. Conversely, TQ was found to enhance the expression of the PDH enzyme [61, 139]. The suppression of the PI3K-AKT/HK2 pathway is associated with a reduction in the tumorigenic capabilities of CRC cells, including wound healing and invasiveness [139].

Previous research has suggested that PGE2 enhances COX-2 expression by activating the EP4/β-catenin pathway, implying that PGE2 regulates p-PI3K, p-AKT, and p-GSK-3β expression in LoVo cells. It has been noted that PGE2 initiates downstream signaling through EP2 and EP4 to induce a variety of biological reactions. The administration of TQ was observed to diminish the increase in COX-2 expression induced by PGE2, and β-catenin significantly influenced the modulation of EP2 and EP4 by PGE2. Hsu et al. reported that TQ successfully inhibited PGE2/EP2/EP4-induced activation of p-Akt/p-PI3K/p-GSK3β/β-catenin/LEF-1/TCF-4 in LoVo cells [141]. Results showed that TQ decreased nuclear translocation of β-catenin/LEF-1/TCF-4 in a concentration-dependent manner which led to downregulation of COX-2. Researchers concluded that COX-2 inhibition led to suppressing cell migration as well as metastasis in vivo [141]. Within the nucleus, β–catenin operates as a transcription factor, forming a complex with TCF/LEF that binds to DNA enhancer sequences. This interaction results in the upregulation of certain genes, including the proto-oncogene c-myc [142]. Research conducted by Lang et al., revealed that TQ translocated β–catenin to the membrane, thereby suppressing c-myc expression in APC^Min^ mice. TQ was found to inhibit the phosphorylation of GSK-3β, likely through the suppression of MEK1/2 rather than PI3K. In untreated colorectal cells, GSK-3β undergoes phosphorylation at the Ser9 position via several pathways (such as Ras-Raf-MEK, PI3K-AKT1, and WNT), rendering it inactive. However, following TQ administration, there was a decrease in GSK-3β Ser9 phosphorylation (which is downstream of Ras, Raf, MEK). This results in β-catenin being relocated to the membrane and a reduction in nuclear c-myc (due to phosphorylation, ubiquitination, and eventual degradation) [143].

TQ has been reported to increase activated (phosphorylated) forms of JNK1/2 and ERK1/2 in DLD-1 cells, likely through ROS production. However, this effect was abolished after 24 h treatment. No alterations were observed in the expression of p-p38 and total p38 protein as well as total JNK and ERK protein in response to TQ [126]. STAT3 is perpetually active in colon cancer and plays a crucial role in cell proliferation by transcriptionally activating pro-survival genes [144]. The treatment of cells with TQ obstructed the continuous phosphorylation of STAT3 at the tyrosine-705 residue and reduced the nuclear localization of p-STAT3. Furthermore, TQ could target EGFR, Src kinase and JAK2. Incubation of 50 μM TQ in HCT-116 cells decreased activated form of JAK2 (p-JAK2) followed by a suppression in p-STAT3 [145]. TQ has been identified as a potent inhibitor of NF-κB, a key cellular transcription factor. At a concentration of 60 µM, TQ was found to suppress the phosphorylation of NF-κB p65 subunit, resulting in the inhibition of its downstream genes including VEGF, c-Myc, and Bcl-2 in COLO205 cells [129].

### The effects of TQ on cell proliferation/cycle

El-Najjar reported that TQ inhibited the proliferation of a panel of CRC cell lines, including HT-29, HCT-116, DLD-1, Lovo, and Caco-2, in a time and concentration-dependent manner. Among these cell lines, Caco-2 was the most sensitive and HT-29 was considered the most resistant to TQ according to their IC_50_ [126]. Gali-Muhtasib et al. [146] found that TQ arrested the cell cycle at G1/S within 24 h/48 h of 60 µM TQ treatment. At elevated doses of TQ (100 µM) and with extended incubation periods, there was a noticeable accumulation of a sub-G1 peak of hypodiploid cells to the left of the G1 peak, along with a corresponding decrease in the S population. The G1/S cell cycle arrest is attributed to p21/WAF1 expression, which prevents transition to the S phase and is enhanced by TQ-induced p53 expression [146].

DLD-1 cells were subjected to a treatment of 40 μM TQ for either 24 or 48 h, and then collected for flow cytometric analysis of DNA content via PI staining. TQ induced a significant rise in the proportion of cells in the preG1 phase of the cell cycle in a time-dependent manner: at 40 μM TQ for 24 h, it increased from 2.5% to 18.8%, and for 48 h, it escalated from 4.0% to 31.2% [126]. Another study used 10 μM and 20 μM TQ and evaluated the effect of TQ following 24 and 72 h. In HCT-116 cells, a 24-h exposure to 20 μM TQ led to a notable build-up of pre-G1 events with a reduction in G1, S, and G2/M events, while no alteration was detected with 10 μM TQ. After 72 h, 10 μM TQ triggered a significant G1/S halt with diminished G2/M events; at 20 μM, the most pronounced disturbance (albeit less than after a 24-h treatment) was the build-up of pre-G1 events [147]. TQ was observed to increase the proportion of HT29 cells in the G2/M phase, while simultaneously reducing the count in the S-phase when compared to untreated cells. Conversely, TQ resulted in a halt at the G0/G1-phase in SW480 cells. This was accompanied by a decline in CCND1 and CCND3 mRNA/protein levels, while an increment was observed in p21 and p27 levels [61] In addition, TQ lessened the expression of STAT3 target gene products, such as survivin, c-Myc, cyclin-D1, -D2, and elevated the expression levels of the cell cycle regulatory proteins p27 and p21 [145]. The growth of HCT-15 cells was observed to be suppressed by TQ in a dose-dependent manner (IC_50_: 82.59 µM). Furthermore, the growth of these cells was adversely affected even when exposed to higher doses of TQ [130]. TQ with ionizing radiation caused G2/M arrest in HT-29 and HCT-116 cells. While radiation (2 Gy) alone led to a minor increase in the proportion of HCT116 cells in the G2/M phase, the combination of radiation with TQ (10 µM and 30 µM) resulted in a significant accumulation of cells in the G2/M phase. Furthermore, there was a notable decrease in the percentage of cells at G0/G1 in HCT116 cells treated with 30 µM TQ and radiation. In HT29 cells, cell cycle arrest was observed in irradiated cells and in cells treated with 60 µM TQ. This effect was more pronounced in cells treated with a combination of TQ and radiation compared to either treatment alone. Interestingly, when combined with radiation, TQ led to a significant increase in the G2/M population from 21% in the control to 26% and 31% at TQ concentrations of 10 µM and 60 µM, respectively [131].

### The effect of TQ on cell death and induction of apoptosis

Treatment with 100 µM of TQ after 24 h reduced Bcl-2 and increased apoptosis in a p53-dependent manner in HCT-116 cells. The suppression of p53 expression curtailed the over-expression of p53 induced by TQ and significantly reduced apoptosis, indicating that p53 is the primary regulator of apoptosis induced by TQ [146]. TQ diminished the expression of the anti-apoptotic proteins Bcl-2 and Bcl-xl, while it amplified the expression of the pro-apoptotic protein Bax in HCT116 cells following treatment with 25 or 50 µM TQ after 24/48/72 h. The administration of TQ to HCT116 cells triggered the cleavage of caspase-9, -7, and -3, and PARP, and heightened the activity of caspase-3. The pre-treatment of cells with a pan-caspase inhibitor z-VAD-fmk nullified the TQ-induced caspase-3 activity, as well as the cleavage of caspase-3 and PARP. Furthermore, obstructing the activation of caspase-3 led to the cessation of TQ-induced apoptosis [145]. TQ instigated a significant increase in apoptosis that was dependent on the concentration after an exposure of 24 h, with most apoptotic events taking place in the late-apoptotic quadrant (A+ /PI+), reaching 10% for 10 µM and 23% for 20 µM. However, after 72 h, there were diminished and non-significant escalations in apoptotic cells observed in these cells, even with a treatment of 20 µM in HCT-116 cells [147].

Treatment with 100 µM of TQ in HT-29 cells led to an increase in the necrosis rate, exceeding 90% after 24 h [148]. TQ was found to increase reactive oxygen species (ROS), specifically the superoxide radical O2^−^ , which subsequently led to DNA damage. This was confirmed by the high expression of γH2AX, a marker of DNA damage [149]. ROS production occurred in both p53^+/+^ and p53^−/−^ HCT-116 cells, but was higher in p53^+/+^ cells. Gali-Muhtasib et al. indicated that p53-induced CHEK1 reduction contributed to apoptosis. Also, CRC clinical samples verified that CHEK1 inhibition was observed in p53 expressing patients rather than p53 null patients [149]. To confirm that p53-induced CHEK1 inhibition is related to caspase-3 dependent apoptosis, HCT-166 p53^+/+^ and p53^−/−^ xenograft mouse models were established, and similar results were obtained [149]. TQ was observed to induce the apoptotic cleavage of PARP, resulting in an 89 kDa fragment at 24 h. However, in the presence of IPA-3, a PAK1 inhibitor, this cleavage was more pronounced and occurred earlier, at 6 h. Intriguingly, the combination of TQ (40 μM) and IPA-3 (10 μM) led to a significant increase in cell death and reduced cell viability by 70% in HCT-116 wt cells [138]. Among CRC cell lines, HT-29 is reported to be the most resistant to TQ-induced apoptosis, while DLD-1 showed TQ-induced early apoptosis. The rise in apoptosis over time due to TQ was further validated by the M30 immunofluorescent pictures, which displayed distinct cytoplasmic indications for the M30 antibody following TQ administration. The M30 cytodeath antibody, which identifies a specific caspase cleavage site within cytokeratin 18, is a characteristic indicator of early apoptosis initiation. Moreover, a 2.5-fold and fourfold surge in caspase-3/7 activity was noted at 24 h and 48 h respectively, following the administration of 40 μM TQ [126]. El-Najjar et al. have stated that oxidative stress is the mechanism through which TQ exerts its anti-cancer and pro-apoptotic effects [126].

Intraperitoneal (i.p.) injection of TQ was found to decrease DMH-induced CRC in female bulb/c mice by promoting caspase-3 and apoptosis. Similar results were obtained in HCT-116 xenograft of this model. Injection of TQ significantly decreased both the count and size of Aberrant Crypt Foci (ACF) at the 10-week mark, with ACF numbers dropping by 86%. Tumor multiplicity was also reduced at the 20-week mark, decreasing from 17.8 in the DMH group to 4.2 in mice injected with TQ. This suppression was observed at the 30-week mark and was long-term; tumors did not re-grow even when TQ injection was discontinued for 10 weeks [150].

### The effect of TQ against cancers based on human studies

A phase 1 randomized, double-blind, placebo-controlled trial was conducted to assess the safety of a black cumin oil formulation containing 5% TQ, administered at a dose of 200 mg per adult per day for a period of 90 days, on healthy participants. The study did not report any serious adverse side effects or significant changes in hematological parameters. Similarly, no significant changes were observed in biochemical parameters related to liver function (ALT, AST, ALP) and renal function (serum creatinine and urea). However, the lipid profile analysis showed a significant reduction in total cholesterol, LDL, VLDL, and triglycerides, albeit within the normal range [151]. A clinical trial was conducted in a randomized, double-blind, placebo-controlled manner to assess the potential benefits of *Nigella sativa* seeds oil as an adjunctive therapy for hypertension, blood sugar regulation, and lipid metabolism. The intervention group were given 2.5 ml of N. sativa seeds oil twice daily for 8 weeks. There was a notable reduction in blood pressure, total cholesterol, low-density lipoprotein, MDA, and FBS levels, along with a significant rise in high-density lipoprotein and Glutathione Reductase levels [152]. In a separate study, it was found that when administered in conjunction with a daily dose of 1000 mg Metformin, both 50 and 100 mg doses of TQ showed a decrease in HbA1c and blood glucose levels. This combination therapy proved to be more effective than the standard treatment of diabetes, which involves administering Metformin alone [153]. A study conducted by Soleymani and colleagues revealed that a hydrogel made from N. sativa had a significant impact on alleviating the symptoms of acne vulgaris. The treatment was also found to be well-tolerated by the patients [154]. Ammar and others concluded that supplementing with black cumin oil, taken as 500 mg soft gel capsules three times a day for a duration of 6 months, has been found to provide additional benefits when used alongside metformin in improving conditions related to Polycystic Ovary Syndrome (PCOS). These benefits include the resumption of regular menstrual cycles, weight loss, alteration in body fat distribution, and the restoration of oxidative balance [155].

According to https://clinicaltrials.gov/, a phase II clinical trial conducted by Nabil investigated the chemopreventive effects of N. sativa. This randomized, controlled study (NCT03208790) enrolled 48 patients with premalignant oral lesions1. The participants were given either a 10 mg N. sativa tablet to the buccal mucosa, a 5 mg buccal N. sativa tablet, or a placebo. The primary outcome measure was the size of the lesion at 3 months post-treatment compared to the initial dimensions. Although the study was completed in 2020, the results have not yet been published [156, 157].

To the best of our current understanding, there have been no clinical studies that specifically investigate the impact of TQ on colorectal cancer.

## Conclusion

Despite notable progress in surgical and chemotherapy procedures, the survival rates of patients with life-threatening diseases such as cancers are still affected by drug resistance and adverse side effects experienced under chemotherapy or radiotherapy [166]. Hence, it is imperative to fortify exploration and development endeavors to enhance the efficacy of prevalent remedial protocols while concurrently curtailing their adverse influence on patient health and standard of living. In this regard, natural agents like TQ have shown immense promise in advancing cancer treatment outcomes. Various studies have illustrated that TQ, through its capability to modulate different signaling pathways, can provide potent anti-cancer properties. The anti-inflammatory and antioxidant features of TQ have been well documented as capable of suppressing colorectal malignancies. Additionally, TQ may affect cellular processes such as apoptosis, angiogenesis, cell cycle, and proliferation, as well as metastasis, thereby enhancing its anticancer effects.

In summary, TQ stands as a promising natural therapeutic agent that can enhance the efficacy of existing cancer treatments while minimizing the associated adverse effects. However, further research is of vital importance in order to acquire a more comprehensive understanding of its exact molecular targets and pathways and maximize its clinical usefulness. These investigations may potentially aid in the development of novel techniques to combat drug resistance and surmount the obstacles presented by chemotherapy and radiotherapy.

## Data Availability

Not applicable.
